# Application of an index derived from the area under a neutrophil curve as a predictor of surgical site infection after spinal surgery

**DOI:** 10.1186/s12893-021-01345-6

**Published:** 2021-09-27

**Authors:** Hiroyuki Inose, Yutaka Kobayashi, Shingo Morishita, Yu Matsukura, Masato Yuasa, Takashi Hirai, Toshitaka Yoshii, Atsushi Okawa

**Affiliations:** 1 Department of Orthopaedic and Trauma Research, Graduate School, Tokyo Medical and Dental University, 1-5-45 Yushima, Bunkyo-ku, Tokyo, 113-8510 Japan; 2 Department of Orthopaedics, Graduate School, Tokyo Medical and Dental University, 1-5-45 Yushima, Bunkyo-ku, Tokyo, 113-8510 Japan

**Keywords:** Predictor, Surgical site infection, I-index, Spinal surgery, Neutrophil curve, Receiver operating characteristic curve

## Abstract

**Background:**

Patients with prolonged and intense neutrophilia after spinal surgery are at high risk of developing surgical site infection (SSI). To date, there is no standard method for the objective assessment of the intensity and duration of neutrophilia. Thus, this retrospective observational study aimed to test a new index (I-index), developed by combining the duration and intensity of neutrophilia, as a predictor of SSI.

**Methods:**

I-index was calculated based on the postoperative neutrophil percentage. A total of 17 patients with SSI were enrolled as cases, and 51 patients without SSI were selected as controls. The groups were matched at a ratio of 1:3 by age, sex, and surgery type. The differences in the I-index were compared between the groups. Moreover, we checked the cumulative I-index (c-I-index), which we defined as the area under the neutrophil curve from postoperative day 1 until the first clinical manifestation of SSI in each case. Furthermore, a cutoff for SSI was defined using the receiver operating characteristic curve.

**Results:**

The median I-index-7, I-index-14, and c-I-index were significantly higher in the SSI group than those in the control group. For a cutoff point of 42.1 of the I-index-7, the sensitivity and specificity were 0.706 and 0.882, respectively. For a cutoff point of 45.95 of the I-index-14, the sensitivity and specificity were 0.824 and 0.804, respectively. For a cutoff point of 45.95 of the c-I-index, the sensitivity and specificity were 0.824 and 0.804, respectively.

**Conclusion:**

We devised a new indicator of infection, i.e., the I-Index and c-I-index, and confirmed its usefulness in predicting SSI.

## Background

In recent years, the efficacy of spinal surgery has been well established [1–5]; as a result, there has been a significant increase in the overall number of spinal surgeries [6]. With this increase, complications such as postoperative infections may arise with some degree of probability [7]. Neutrophils have been reported to play an important role in the defense against these bacterial infections [8]; hence, their number increases during infection [9]. Previous studies have found that older age, instrumentation surgery, smoking, alcohol drinking, preoperative hemoglobin, preoperative albumin, and the National Nosocomial Infection Surveillance (NNIS) index may affect the occurrence of postoperative surgical site infection (SSI) [10–15]. In addition, postoperative neutrophilia is considered a significant predictor of SSI [7, 16]. Accordingly, the presence of infection is strongly suspected when high neutrophil levels persist over an extended time. Despite this reported close association of prolonged neutrophilia with the development of SSI, to our knowledge, no tools have been specifically developed for the measurement of the intensity and duration of neutrophilia.

The D-index, which is used as a tool for predicting invasive fungal infections, is reportedly based on a graph that shows the absolute neutrophil count during neutropenia and is calculated as the area over the neutrophil curve [17, 18]. Based on this idea, we conceived the infection index (I-index) for predicting SSI. This study aimed to introduce the I-index and evaluate the usefulness of the I-index as a predictor of SSI in patients after spinal surgery.

## Methods

### Defining the I-index and c-I-index

Because postoperative long-lasting intense neutrophilia suggests SSI occurrence, we hypothesized that the area under the neutrophil curve above a neutrophil percentage of 70%, the cutoff value of neutrophilia, would represent the duration and intensity of neutrophilia. In this study, the cutoff value of the neutrophil percentage, which defines neutrophilia, was 70% because the standard upper limit of the neutrophil percentage in the peripheral blood was 70% [19–22]. This observed area under the curve was termed the I-index and calculated using the trapezoidal method. The I-index-7 was calculated based on a graph that plotted the neutrophil percentage over 7 postoperative days. The I-index-14 was calculated based on a graph that plotted the neutrophil percentage over 14 postoperative days. Finally, the cumulative I-index (c-I-index) was calculated as the area under the curve starting from postoperative day 1 until the day when the SSI was diagnosed. Accordingly, if the neutrophil percentage is greater than 70% and infection is evident after 14 days postoperatively, the c-I-index value is higher than the I-index-14 value. Conversely, if the neutrophil percentage increases transiently after surgery and falls below 70% within 14 days and no infection occurs thereafter, the c-I-index value will be the same as the I-index-14 value.

### Parameters used for the calculation of the I-index-7, I-index-14, and c-I-index

The I-index is calculated as the area surrounded by the neutrophil percentage curve and the horizontal line at a neutrophil percentage of 70%, to evaluate both the severity and duration of neutrophilia. In this study, the calculation was started on postoperative day 1, and the measurement period was up to postoperative day 7 (I-index-7), postoperative day 14 (I-index-14), or until the day when the SSI was diagnosed (c-I-index).

Accordingly, the I-index is calculated using the trapezium rule:

$${\text{I - index}} = \mathop \sum \limits_{i = 2}^{n} \left( {t_{i} { } - t_{i - 1} } \right)\frac{{N_{i - 1} + N_{i} }}{2}$$

where (t_i_–t_i-1_) is the time interval (days) between two consecutive neutrophil percentages and N_i_ and N_i-1_ are the respective neutrophil percentages minus 70 at times t_i_ and t_i-1_. To simplify the calculation, if N_i_ or N_i-1_ is less than 0, the value is considered 0. For two neutrophil percentages, we have one trapezium; hence, for n measures, we will have n-1 trapezia.

**Fig. 1:**
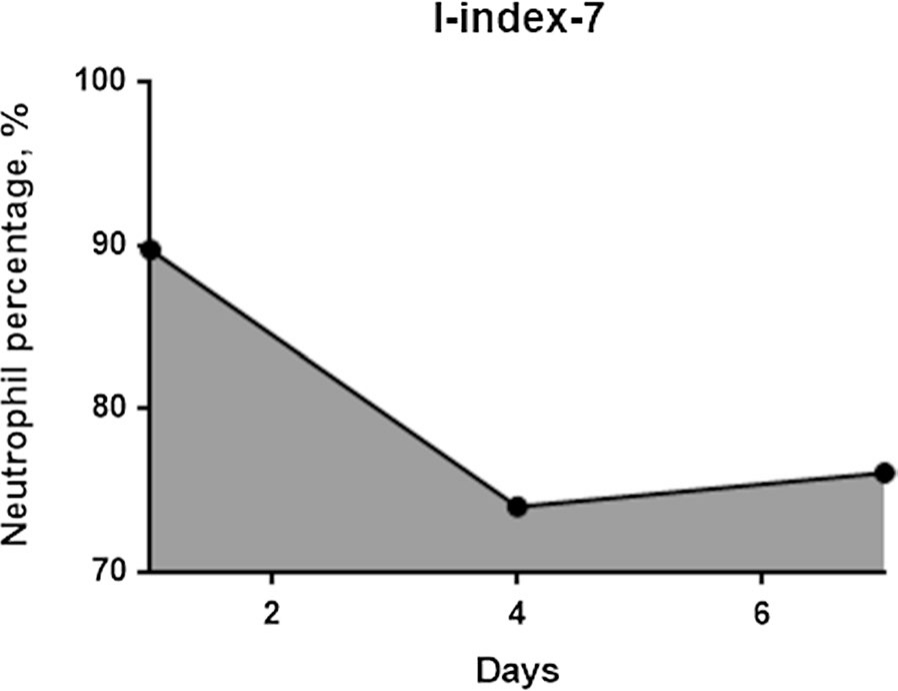
Graph of the neutrophil percentage versus the number of postoperative days. The area under the neutrophil curve above the 70% neutrophil percentage is the I-index-7

As an example, the I-index-7 for patient 1 with neutrophil percentages of 89.7% on day 1, 74% on day 4, and 76.1% on day 7 is computed as the sum of the area of two trapezia because we have three neutrophil percentages (n = 3) (Fig. 1).

Hence,

$${\text{I}} - {\text{index}} - 7 = {\mkern 1mu} \left\{ {1/2\left[ {\left( {89.7 - 70} \right){\mkern 1mu} + {\mkern 1mu} \left( {74 - 70} \right)} \right]{\mkern 1mu} \times {\mkern 1mu} \left( {4 - 1} \right)} \right\}{\mkern 1mu} + {\mkern 1mu} \left\{ {1/2\left[ {\left( {74 - 70} \right){\mkern 1mu} + {\mkern 1mu} \left( {76.1 - 70} \right)} \right]{\mkern 1mu} \times {\mkern 1mu} \left( {7 - 4} \right)} \right\}{\mkern 1mu} = {\mkern 1mu} \left[ {1/2\left( {19.7 + 4} \right){\mkern 1mu} \times 3} \right]{\mkern 1mu} + {\mkern 1mu} \left[ {1/2\left( {4 + 6.1} \right){\mkern 1mu} \times 3} \right]{\mkern 1mu} = 35.55 + 15.15 = 50.{\text{7 days}} \cdot {\text{percentage}}$$

**Fig. 2:**
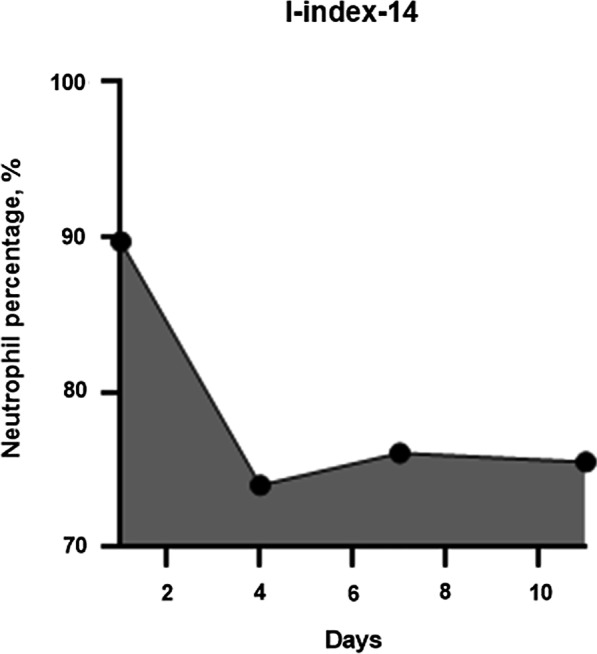
Graph of the neutrophil percentage versus the number of postoperative days. The area under the neutrophil curve above the 70% neutrophil percentage is the I-index-14

The I-index-14 for patient 1 with neutrophil percentages of 89.7% on day 1, 74% on day 4, 76.1% on day 7, and 75.5% on day 11 is computed as the sum of the area of three trapezia because we have four neutrophil percentages (n = 4) (Fig. 2).

Hence,

$${\text{I}} - {\text{index}} - {14 } = \, \left[ {{1}/{2}\left( {{19}.{7 } + { 4}} \right) \, \times { 3}} \right] \, + \, \left[ {{1}/{2}\left( {{4 } + { 6}.{1}} \right) \, \times { 3}} \right] \, + \, \left[ {{1}/{2}\left( {{6}.{1 } + { 5}.{5}} \right) \, \times { 4}} \right] \, = { 35}.{55 } + { 15}.{15 } + { 23}.{2 } = { 73}.{\text{9 days}} \cdot {\text{percentage}}$$

**Fig. 3:**
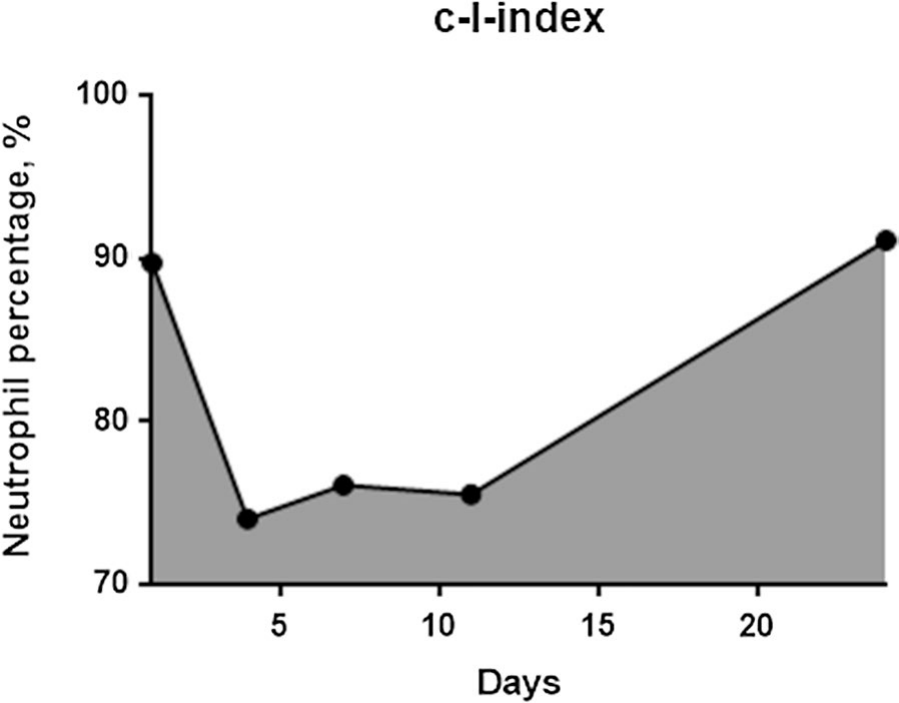
Graph of the neutrophil percentage versus the number of postoperative days. The area under the neutrophil curve above the 70% neutrophil percentage is the c-I-index

The c-I-index for patient 1 with neutrophil percentages of 89.7% on day 1, 74% on day 4, 76.1% on day 7, 75.5% on day 11, and 91.1% on day 24 is computed as the sum of the area of four trapezia because we have five neutrophil percentages (n = 5) (Fig. 3).

Hence,

$${\text{c}} - {\text{I}} - {\text{index }} = \, \left[ {{1}/{2}\left( {{19}.{7 } + { 4}} \right) \, \times { 3}} \right] \, + \, \left[ {{1}/{2}\left( {{4 } + { 6}.{1}} \right) \, \times { 3}} \right] \, + \, \left[ {{1}/{2}\left( {{6}.{1 } + { 5}.{5}} \right) \, \times { 4}} \right] \, + \, \left[ {{1}/{2}\left( {{5}.{5 } + { 21}.{1}} \right) \, \times { 13}} \right] \, = { 35}.{55 } + { 15}.{15 } + { 23}.{2 } + { 172}.{9 } = { 246}.{\text{8 days}} \cdot {\text{percentage}}$$

### Patient selection and assessment of the I-index as a predictive factor for SSI

This study was conducted in the Department of Orthopedic Surgery at the Tokyo Medical and Dental University Hospital in Japan between April 2014 and February 2018 on consecutively hospitalized patients who underwent spinal surgery. A total of 496 patients were selected. Among them, 17 cases were enrolled as SSI cases. We then selected patients without SSI as the controls after they were matched to an SSI subject at a ratio of 1:3 according to age, sex, and surgery type using propensity score.

Prophylactic antibiotics were administered during and after surgery for a total of 48 h, according to the Japanese Orthopaedic Association Clinical Practice Guideline on the Prevention of Surgical Site Infections in Bone and Joint [23]. The patients received cefazolin (1.0 g, intravenous) 30 min before skin incision. The antibiotic was administered intravenously in additional doses of 1.0 g every 3 h during and every 12 h after surgery. SSI was diagnosed based on the definition provided by the Centers for Disease Control and Prevention as superficial, deep, or organ-space infections occurring within 1 year postoperatively in the case of instrumentation surgery and within 30 days postoperatively in the case of decompression surgery [24]. In addition, the presence of SSI was confirmed by reoperation or histopathological or radiological investigation.

The neutrophil percentage and lymphocyte percentage and count were collected. We also recorded the following: number of segments operated on; surgery type; history of diabetes mellitus; current smoking; current alcohol drinking; NNIS index; preoperative hemoglobin and albumin; uses of steroids, immunosuppressive agents, and biological antirheumatic drugs; operative time; intraoperative blood loss; and number of blood draws. The NNIS Index evaluates the risk of SSI according to American Society of Anaesthesiologists classification of physical health; the Altemeier wound contamination class and surgical duration. Each factor is rated 0 or 1 in terms of presence or absence, respectively, of the risk factor [25].

### Statistical analysis

Power analysis with a significance of 0.05, power of 0.80, an assumed mean of 26, and an assumed SD of 20 for I-index-7 in the non-SSI group was used to calculate the sample size for this study. A minimum of 45 non-SSI patients was required to detect a significant difference in the I-index-7 value between SSI and non-SSI groups. Accordingly, we decided to enroll 51 patients into the non-SSI group (SSI:non-SSI = 1:3). Among the baseline variables, age, sex, and surgery type were matched between the SSI and non-SSI groups in a 1:1 ratio with a caliper of 0.20 using propensity scores derived from comparisons of these variables. The matching process was performed using the jmpjapan.addin.matching.jmpaddin package developed for JMP (SAS Institute, Cary, NC, USA). The matching process was continued until there were 51 patients in the non-SSI group. Differences between the SSI and non-SSI groups were analyzed. After assessing data normality with the Shapiro–Wilk test, continuous variables with normally distributed data and those with skewed distribution were analyzed using Welch’s t-test and Mann–Whitney *U* test, respectively. Fisher’s exact test was used for nominal variables. We then performed analyses with the presence or absence of SSIs as a dependent variable. A receiver operating characteristic (ROC) curve was generated to assess the cutoff point, sensitivity, and specificity of the I-index and c-I-index for predicting SSI. JMP version 12 was used for statistical analyses, while Stata 16 (Stata Corporation, College Station, TX, USA) was used for the power analysis. A p-value of < 0.05 was considered significant.

## Results

### Demographics

The SSI group included 10 male and 7 female patients, whereas the non-SSI group included 30 male and 21 female patients (Table 1). The mean ages at the surgery in the SSI and non-SSI groups were 72.2 (standard deviation (SD), 9.2) and 72.1 (SD, 8.9) years, respectively. The operational features in the SSI and non-SSI groups were as follows: mean operative time, 212 (SD, 109) and 219 (SD, 100) min, and mean intraoperative blood loss, 230 (SD, 162) and 298 (SD, 316) mL, respectively. In particular, the 17 patients with SSI and 51 controls had a similar mean number of blood draws over the 14 postoperative days (4.5 vs. 4.3; p = 0.16). The number of operated segments was significantly higher in the SSI group (p = 0.01); however, there was no significant difference between the two groups in the other background factors (Table 1). Regarding the treatment for SSI, debridement was performed in 13 of the 17 SSI patients.

**Table 1:** Clinical characteristics of the study patients

	SSI group	Non-SSI group	p
Age (SD), years	72.2 (9.2)	72.1 (8.9)	0.99
Sex	Male 10, Female 7	Male 30, Female 21	> 0.99
No. of operated segments (SD)	3.2 (1.8)	2.2 (1.9)	0.01*
Surgery type, n	Decompression 6 Fusion 11	Decompression 18 Fusion 33	> 0.99
History of diabetes mellitus, n (%)	3 (17.6)	7 (13.7)	0.70
Smoking, n (%)	1 (5.9)	6 (11.8)	0.67
Alcohol, n (%)	4 (23.5)	22 (43.1)	0.25
NNIS risk index (SD)	0.76 (0.75)	0.53 (0.50)	0.29
Preoperative hemoglobin (SD), g/dL	13.0 (2.0)	13.1 (1.7)	0.97
Preoperative albumin (SD), g/dL	3.8 (0.6)	4.0 (0.3)	0.27
Preoperative Neutrophil percentage (SD)	67.9 (12.2)	61.3 (9.4)	0.05
Use of steroids, n (%)	2 (11.8)	2 (3.9)	0.26
Use of immunosuppressive agents, n (%)	1 (5.9)	1 (2.0)	0.44
Use of biological anti-rheumatic drugs, n (%)	1 (5.9)	0 (0)	0.25
Operative time (SD), minutes	212 (109)	219 (100)	0.68
Blood loss (SD), ml	230 (162)	298 (316)	0.78
Number of blood draws during 14 days after surgery (SD), n	4.5 (0.5)	4.3 (0.6)	0.16

Values are presented as mean ± standard deviation or number (%)

*Statistically significant (p < 0.05). *SSI* surgical site infection, *SD* standard deviation

### Evaluation of indexes for the prediction of SSI

The mean I-index-7 and I-index-14 were significantly higher in the SSI group than in the controls (p = 0.002 and 0.001). Furthermore, not surprisingly, the c-I-index was significantly higher in the SSI group (p < 0.0001) (Table 2). The number and percentage of lymphocytes, which have been reported to be useful as early predictive markers of SSI [26, 27], were significantly lower in the SSI group at 6–7 days postoperatively (p = 0.01 and 0.001).

**Table 2:** Comparison of I-index and lymphocyte count and percentage between groups

	SSI group	Non-SSI group	p
I-index-7 (SD)	47.8 (28.1)	25.4 (14.4)	0.002*
I-index-14 (SD)	71.4 (44.2)	32.6 (23.1)	0.001*
c-I-index (SD)	113.6 (92.7)	32.6 (23.1)	< 0.0001*
Lymphocyte count at 3–4 days postoperatively (SD)	1070 (328)	1248 (428)	0.10
Lymphocyte percentage at 3–4 days postoperatively (SD)	15.2 (5.7)	18.7 (7.8)	0.16
Lymphocyte count at 6–7 days postoperatively (SD)	1078 (384)	1396 (482)	0.01*
Lymphocyte percentage at 6–7 days postoperatively (SD)	17.2 (7.6)	25.0 (7.4)	0.001*

Values are presented as mean ± standard deviation

*Statistically significant (p < 0.05). *SSI* surgical site infection, *SD* standard deviation

**Fig. 4:**
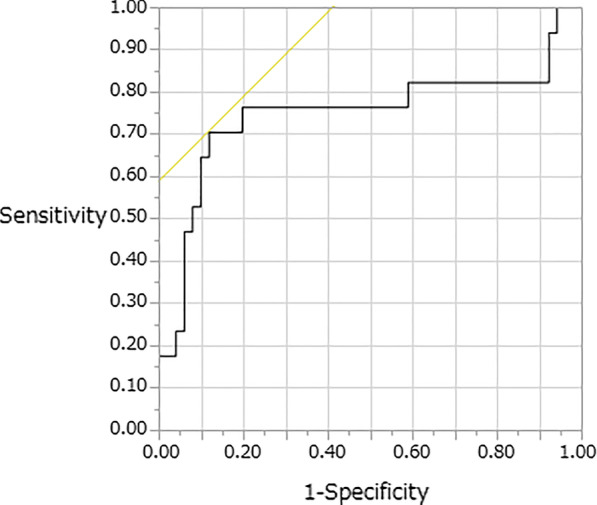
Identification of the cutoff value of the I-index-7 for surgical site infection using the receiver operating characteristic analysis; area under the curve = 0.751; p = 0.0002

**Fig. 5:**
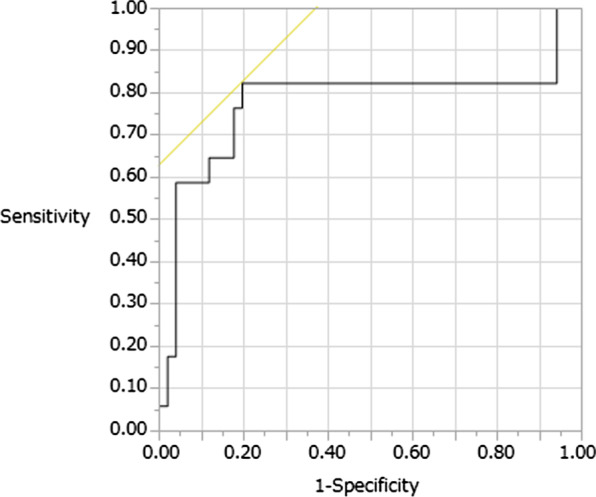
Identification of the cutoff value of the I-index-14 for surgical site infection using the receiver operating characteristic analysis; area under the curve = 0.776; p < 0.0001

**Fig. 6:**
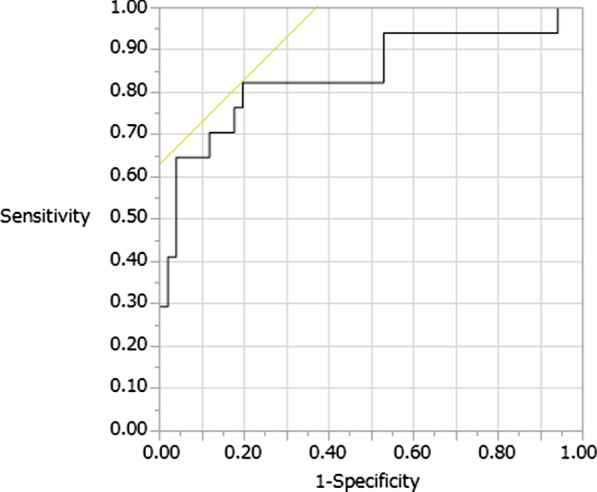
Identification of the cutoff value of the c-I-index for surgical site infection using the receiver operating characteristic analysis; area under the curve = 0.842; p < 0.0001

The cutoff value of the I-index with its relevant sensitivity and specificity was derived by the ROC analysis. The ROC curve showed good sensitivity and specificity of the I-index-7, I-index-14, c-I-index, and lymphocyte count and percentage at 6–7 days postoperatively for SSI (area under the ROC curve, 0.751, 0.776, 0.842, 0.689, and 0.785). The optimum cutoff value of the I-index-7 for predicting SSI after spinal surgery according to the Youden index was 42.1. At this level, the sensitivity and specificity were 0.706 and 0.882, respectively (Fig. 4). The optimum cutoff value of the I-index-14 for predicting SSI after spinal surgery according to the Youden index was 45.95. At this level, the sensitivity and specificity were 0.824 and 0.804, respectively (Fig. 5). The optimum cutoff value of the c-I-index for predicting SSI according to the Youden index was 45.95. At this level, the sensitivity and specificity were 0.824 and 0.804, respectively (Fig. 6). The optimum cutoff value of the lymphocyte count at 6–7 days postoperatively for predicting SSI according to the Youden index was 1074. At this level, the sensitivity and specificity were 0.588 and 0.726, respectively. The optimum cutoff value of the lymphocyte percentage at 6–7 days postoperatively for predicting SSI according to the Youden index was 19.8%. At this level, the sensitivity and specificity were 0.824 and 0.745, respectively.

## Discussion

In this study, we showed that the neutrophil curve, in the form of the I-index, was associated with the development of SSI. The calculation for the I-index is simple since it requires only the neutrophil percentage. We showed that the I-index was higher in the SSI group than in the non-SSI group. In previous reports, the possibility of SSI was determined based on values at a certain time point, such as the neutrophil percentages at 4 and 6–7 days postoperatively and lymphocyte count and percentage at 4 and 7 days postoperatively [7, 27]. However, in the I-index, the neutrophil percentage at different time points can be used as a single parameter to predict the likelihood of SSI. By reflecting the state of neutrophils at different time points, the prediction accuracy can be improved. Indeed, in this study, the diagnostic accuracy of the I-index-14 and c-I-index was higher than that of the I-index-7 based on the area under the ROC curve. Interestingly, according to Takahashi et al., the number and percentage of lymphocytes were not significantly different between the SSI and control groups at 2 and 3 weeks postoperatively [26]. Another advantage of using the I-index is that it can be used to predict the occurrence of SSI by theoretically estimating the number of neutrophils in the course of the disease, without the need for frequent blood sampling.

Similar to the results of previous studies, the lymphocyte percentage at 6–7 days postoperatively was useful for the early prediction of SSIs in the present study [26, 27]. Considering the use of I-index-7 and lymphocyte percentage at 6–7 days postoperatively for the early detection of SSI, I-index-7 had higher specificity and lymphocyte percentage had higher sensitivity. Therefore, instead of using only one of them, it may be better to use these two predictors together to help in the early detection of SSI.

According to the results of our study, if the I-index-7 is greater than 42.1 during the postoperative follow-up period of 7 days or the I-index-14 is greater than 45.95 during the postoperative follow-up period of 14 days, development of SSI is possible. Since neutrophils are a nonspecific marker of inflammation, the I-indexes can also be increased by respiratory tract infections and urinary tract infections. Therefore, if the I-index is high after surgery, the wound should be examined closely and frequently to determine whether the infection originates from the wound or another site. Further, if the patient has a fever, redness in or around the wound, or pus exuding from the wound, a more intensive diagnostic approach such as culture of the exudate, magnetic resonance imaging, and blood culture is recommended. In addition, the use of biomarkers such as procalcitonin, C-reactive protein, or presepsin may be used to confirm the presence of an infection, since the combination of different tests may increase their positive predictive value [28]. After the definitive diagnosis of SSI, surgeons should start antibiotic administration, that is, targeted therapy, and/or perform debridement. Regular and careful follow-up is recommended even if the initial test results do not indicate SSI.

In addition, in this study, we found that the cutoff value of the c-I-index was 45.95. Therefore, if the c-I-index is greater than 45.95 after 14 days postoperatively, there is a possibility of developing SSI. In our case group, invasive debridement was performed in 13 out of 17 cases of SSI. Conversely, four cases improved with antibiotics alone. Early-stage infection may be manageable with antibiotics alone as the number of bacteria would be predictably low. Indeed, the D-index has been used to determine whether or not to administer empiric antibiotics in febrile neutropenia, and its usefulness has been confirmed in a prospective randomized study [29]. Nonetheless, the use of the I-index as a tool for the pre-emptive anti-SSI therapy needs further research. We are considering a therapeutic strategy involving early imaging and pre-emptive antibiotic administration for patients with a high I-index or c-I-index; however, this strategy needs to be investigated further.

In this study, the number of operated segments was significantly higher in the SSI group than in the non-SSI group, but no significant differences were found in other factors between the two groups. However, the matching was based on age, gender, and surgery type. In particular, older age and instrumentation surgery are considered risk factors for SSI [15]. Therefore, the lack of significance in this study does not mean that these factors are not associated with SSI.

This study had several limitations. First, the sample size was relatively small and used simply to determine whether the I-index is useful for predicting SSI. The post hoc power analysis showed that the sample size of this study had an 84.8%, 90.8%, and 91.8% power in the prediction of SSI using the I-index-7, I-index-14, and c-I-index, respectively. These percentages are more than the 80% threshold conventionally regarded as the minimum acceptable power for a study to be valid. Second, because the number and timing of blood draws are slightly different among institutions, the appropriate cutoff values for the I-index and c-I-index to predict SSI may further vary among institutions. We calculated the I-index over 7 and 14 postoperative days, although the accuracy of the prediction for SSI could be improved by considering a longer follow-up period or increasing the frequency of drawing blood. Some facilities do not admit patients for 7 days after spinal surgery. Accordingly, it may not be possible to draw blood as often as in the current study in such facilities. This may hinder the application and generalization of the I-index. Ideally, we suggest that the I-index be assessed with the same number of blood draws and follow-up period as in our study. However, if the number of blood draws and days of observation differ, the modified I-index can be calculated according to the number of blood draws and days of observation at each facility. Further studies are warranted to overcome these limitations and validate the results of our study. We anticipate various refinements to our I-index based on further research.

In conclusion, the I-index and c-I-index were associated with the development of SSI after spinal surgery. The results of the current study suggest that these simple tools can be useful for the early detection of SSI in patients who have undergone spinal surgery.

## Data Availability

The datasets generated and/or analyzed during the current study are not publicly available due to conditions of ethical approval but are available from the corresponding author on reasonable request**.**

## Abbreviations

SSI: Surgical site infection
I-index: Infection index
ROC: Receiver operating characteristic
